# Basal insulin intensification with GLP-1RA and dual GIP and GLP-1RA in patients with uncontrolled type 2 diabetes mellitus: A rapid review of randomized controlled trials and meta-analysis

**DOI:** 10.3389/fendo.2022.920541

**Published:** 2022-09-08

**Authors:** Giuseppe Lisco, Anna De Tullio, Olga Disoteo, Vincenzo De Geronimo, Giuseppina Piazzolla, Giovanni De Pergola, Vito Angelo Giagulli, Emilio Jirillo, Edoardo Guastamacchia, Carlo Sabbà, Vincenzo Triggiani

**Affiliations:** ^1^ Interdisciplinary Department of Medicine, Section of Internal Medicine, Geriatrics, Endocrinology and Rare Diseases, University of Bari “Aldo Moro”, School of Medicine, Policlinico, Bari, Italy; ^2^ Diabetology Unit, ASST Grande Ospedale Metropolitano Niguarda, Milan, Italy; ^3^ Unit of Endocrinology, Policlinico Morgagni CCD, Catania, Italy; ^4^ National Institute of Gastroenterology, Saverio de Bellis, Research Hospital, Bari, Italy; ^5^ Department of Basic Medical Sciences, Neuroscience and Sensory Organs, School of Medicine, University of Bari, Bari, Italy

**Keywords:** GIP, GLP-1, tirzepatide, basal insulin, type 2 diabetes, obesity, body weight, hypoglycemia

## Abstract

Tirzepatide, a dual agonist of Glucose-Dependent Insulinotropic Polypeptide (GIP) and Glucagon-Like Peptide 1 (GLP-1) receptors, improved glucose control and reduced body weight in different therapeutic approaches. Herein, we overviewed the role of GIP and GLP-1 in the pathophysiology of type 2 diabetes and systematically reviewed the efficacy and safety of injectable incretin-based therapy added to basal insulin in light of the results of the SURPASS-5 trial. We identified eleven randomized clinical trials. GLP-1 receptor agonists (GLP-1RAs) or Tirzepatide added to basal insulin than rigorously titrated basal insulin significantly ameliorates glucose control (Δ HbA_1c_ = -1%, 95% CI -1.25; -0.74, I^2^ 94%; Δ FPG = -14.6 mg/dL, 95% CI -21.6-; -7.6, I^2^ 90%; chance to achieve HbA_1c <_7% = RR 2.62, 95% CI 2.10; 3.26, I^2^ 89%), reduces body weight (Δ = -3.95 kg, 95% CI -5.1, -2.79, I^2^ 96%) without increasing the risk of hypoglycemia (RR = 1.01, 95% CI 0.86; 1.18, I^2^ 7.7%). Tirzepatide provides an impressive weight loss exceeding that observed with GLP-1RAs. Injectable incretin-based therapy plus basal insulin remains a potent and safe therapeutic approach in uncontrolled type 2 diabetes patients previously treated with basal insulin alone. Tirzepatide is expected to ameliorate the management of “diabesity” in this usually difficult-to-treat cluster of patients.

## Background

The endocrine role of the small intestine in the regulation of pancreatic secretion and glucose control dates back to the early 1900s when mechanistic studies demonstrated that chloride acid administration in the duodenum and jejunum stimulated pancreatic secretion, and intestine extracts administered to patients with type 2 diabetes mellitus (T2D) reduced glycosuria (1, 2). After numerous unsuccessful attempts to purify these substances, in 1930, the Belgian La Barre did it. Particularly, La Barre and coworkers isolated two fractions from animal duodenum that exhibited two different activities: the first stimulated the exocrine pancreas, while the second reduced glucose levels without affecting exocrine pancreas secretion. It was hypothesized that an intestinal peptide could regulate glucose levels by enhancing insulin secretion, hence introducing the concept of “intestine secretion insulin” or incretin as a possible therapeutic strategy in T2D (3).

McIntyre and Elrick formally demonstrated the previously hypothesized mechanism in the 1960s, both showing that significantly higher insulin response was observed after oral rather than intravenous glucose load at comparable blood glucose concentrations (4, 5). This phenomenon was defined as the incretin effect. Perley and Kipnis demonstrated that the incretin effect was responsible for at least 50%, and possibly two-thirds, of glucose-related insulin response after oral glycemic load in healthy individuals. At the same time, the contribution of other gastrointestinal peptides (e.g., cholecystokinin, secretin, and gastrin) in mediating the incretin effect was ruled-out and, in the early 1970s, Brown and Dupré isolated a new gastrointestinal peptide that affected gastric motility and secretion and induced a relevant insulin release in response to hyperglycemia but not euglycemia (6). Given these biological properties, the novel peptide was named Gastric Inhibitory Polypeptide, successively baptized as Glucose-dependent Insulinotropic Polypeptide or GIP (6).

In the 1960s, the widespread use of a new radioimmunoassay method led to the incidental discovery of glucagon-like immunoreactivity in the colonic mucosa, attributed to the so-named gut-glucagon successively identified as GIP. Although GIP was identified as a first contributor to the incretin effect, there was evidence that this effect persisted in experimental models of direct GIP antagonism. Hence, it was supposed that other gastrointestinal peptides could have contributed to the glucose-related insulin release. In the early 1980s, Bell and coworkers provided the first sequencing of the mammalian glucagon gene and demonstrated the existence of the so-named Glucagon-Like Peptide-1 (GLP-1) located in the encoding sequence of pro-glucagon (3). The incretin effect of GLP-1 was later demonstrated by Habener and Holst (3).

## GIP and GLP-1: an overview

GIP is a gastrointestinal polypeptide composed of 42 amino acids secreted by K cells in the duodenum and jejunum mucosae. GLP-1 comprises 31 amino acids and is secreted at the level of L cells in the terminal ileum and colonic mucosae (3). GIP and GLP-1 receptors belong to the seven transmembrane G protein-coupled receptors and are abundantly expressed on the human ß-cell membrane (7, 8). GIP receptor (GIPR) has also been found on human α-, δ- and γ-cells, while GPL-1 receptor (GLP-1R) is expressed in a minority of human α- and δ-cells. GIPR has also been identified in human subcutaneous and visceral white adipose tissue and cultured osteoblasts. GLP-1R is moderately expressed in several brain areas in non-human primates, such as the nucleus accumbens, substantia nigra, and the amygdala, thus playing a role in regulating food intake. A weak expression of both receptors has been described in human cardiomyocytes (including sino-atrial node), intestine, pneumocytes, isolated endothelial cells, and seminiferous tubules in mice (7, 8).

Basal GIP and GLP-1 concentrations are very low at fast but increase remarkably after meal ingestion. GIP release is more evident after carbohydrates and lipids consumption (9), while GLP-1 release is enhanced by amino acids such as glutamine (7). GLP-1 release appears to be diphasic (10). The first phase of GLP-1 release occurs 10-15 minutes after meal intake. It is probably mediated by neuroendocrine mechanisms that include brainstem vagal stimulation (an anticipatory mechanism), mechanical stretching of the stomach and duodenum due to food transit, GIP release, and gastrin-related peptide from gastric and duodenal mucosae that, in turn, activate vagal efferences to distal intestine. The intestinal transit stimulates the second wave of GLP-1 release throughout L-cells in the distal intestine (10). GIP release occurs rapidly after meal intake and persists over time (up to four hours), with a serum and intestinal peak of concentration two hours later (11).

Insulin suppresses GIP secretion resulting from negative feedback, while several cytokines, including interleukins 1 and 6, enhance GIP and GLP-1 release (7, 8). Dipeptidyl-peptidase 4 (DPP-IV) activity is particularly intense, and the enzyme degrades both GIP and GLP-1 a few minutes after their release. Therefore, native GIP and GLP-1 have a short half-life (2-5 minutes), and almost all the action of these incretins takes place in the intestine and the portal vein system. However, a small amount (5-10%) of both incretins reaches the systemic circulation, where GIP and GLP-1 may carry out their pleiotropic effects (e.g., cardiovascular level). Therefore, a large amount of circulating GLP-1 is represented by its inactive peptide GLP-1 (8–35) with a null or mild antagonistic effect compared to the intact GLP-1.

A small amount of GLP-1 originates in the pancreas after the local breakdown of its precursor pro-glucagon. Both systemic and locally originated GLP-1 reach pancreatic islets and regulate insulin, glucagon, and somatostatin secretion (7). Both GIP and GLP-1 enhance insulin secretion in a glucose-dependent manner. Hyperglycemia is a permissive factor to GIP and GLP-1 action as the more the glucose concentration, the great the magnitude of ß-cells membrane depolarization and vice versa (7).

The underlying mechanisms by which GLP-1 suppresses glucagon secretion are still debated and include: 1) a direct GLP-1-mediated suppression of glucagon release, but this hypothesis appears controversial especially considering human α-cells that do not express GLP-1R; 2) an indirect effect due to GLP-1-mediated insulin release; 3) an indirect effect due to GLP-1-mediate somatostatin release (7, 8, 11, 12). GIP enhances glucagon secretion, and this action is directly mediated by GIPR agonism (via intracellular Protein Kinase A pathway) since GIPR is abundantly expressed on human α-cells (7, 8). This effect was described in experimental conditions in response to hypoglycemia and euglycemia but not hyperglycemia, suggesting that GIP probably does not contribute to hyperglucagonemia in T2D (7, 8).

The incretin effect is dampened in different metabolic conditions such as for overweight/obesity, insulin resistance, impaired glucose tolerance, and T2D, in which both incretin secretion and effect are disturbed (11). A GIP resistance has been described in T2D due to diminished GIPR expression or accelerated GIPR clearance. Higher or inappropriately normal levels of GIP are detected in T2D patients also during the fast, and the fine regulation of insulin and glucagon release in response to different glycemic levels is problematic. On the other side, GLP-1 response is preserved in T2D, but GLP-1 secretion in response to meal consumption weakens over time, making GLP-1-based therapy an effective strategy to manage hyperglycemia as replacement therapy (7, 13, 14).

GIP and GLP-1 exhibit extra glycemic effects. GLP-1 slows gastric and duodenal emptying, delays meal digestion, and reduces postprandial glycemic excursion, which, in turn, is an essential target of glycemic control in T2D (10). GLP-1 regulates gastrointestinal motility by several mechanisms that include direct activation of vagal efferences (peripheral pathway), cerebral regulation (central pathway), and modulation of local myenteric ganglion activity that controls circular muscle movement, such in a way to reduce evocated peristaltic bustle (10). In addition, GLP-1 reduces food intake by modulating the activity of cerebral areas involved in appetite control, such as the area postrema, amygdala, nucleus accumbens, and substantia nigra (7). The role of GIP in regulating food intake appears controversial. Mechanistic studies revealed that GIPR is expressed in cerebral areas that regulate food intake, such as the area postrema, arcuate, and paraventricular nuclei and GIPR expression overlaps GLP-1R expression (15). GIP signaling is expected to directly induce the activation of anorexigenic areas or facilitate GLP-1 action (15). Conversely, GIP seems not to affect gastrointestinal motility when administered in physiologic and supraphysiologic doses (7, 16), and no evidence, to date, has been provided for GIP to facilitate GLP-1-induced gastric empty delay (7). Incretins could reduce intestinal water resorption, and this mechanism is responsible for diarrhea, a well-recognized adverse effect of GLP-1 receptor agonists (GLP-1RAs).

GIP increases insulin sensibility of white adipose tissue, consequently improving lipid storage and ameliorating lipid profile (mainly triglycerides) and skeletal muscle (7). GIP-mediated actions appear to be additional than those exerted by GLP-1, which improves insulin sensibility in the liver (7).

GIP may stimulate osteoblast activity and bone remodeling, thus providing a possible reduction of fracture risk as similarly observed with GLP-1.

Other effects have been described for GLP-1 (and possibly GIP, with scantier evidence), including anti-inflammatory and vasoactive proprieties (17).

## Dual GIP and GLP-1 receptor agonist: focus on Tirzepatide

Gut-based therapy has become an attractive therapeutic approach for improving glucose control and the burden of related-comorbidity in T2D (18). In this field, GLP-1RAs are demonstrated to allow significant glycemic and extra-glycemic benefits when administered to this cluster of patients (19). Inversely, isolated GIP therapy is not currently deemed as medical therapy for T2D because of pathophysiological considerations. A marked GIP resistance has been described in T2D just in the early phase of the disease, and the contribution of pharmacological GIP administration to repristinate the incretin effect is scanty. In addition, despite controversial evidence, GIP may stimulate glucagon release even in hyperglycemic conditions, as observed in prediabetes and T2D, thus potentially contributing to a further increase of glycemia (20). However, GIP resistance is rapidly reversible when euglycemia is restored, and, in this setting, GLP-1 may significantly improve GIP signaling thanks to additive mechanisms. GLP-1 suppresses glucagon secretion and may completely hide GIP-related hyperglucagonemia in euglycemia but not in hypoglycemia hence maintaining counterregulatory response to hypoglycemia unaltered (20). Moreover, both GIP and GLP-1 repristinate the incretin effect, reduce appetite, and affect caloric intake by some synergic and affine mechanisms that could significantly contribute to achieving more therapeutic objectives (i.e., obesity) in people with T2D (20, 21).

Evidence suggests that dual agonists of GIPR and GLP-1RA can represent an attractive therapeutic opportunity shortly (20, 22–25). From this point of view, the biotechnological goal is to synthesize chimeric molecules that can activate both receptors alternatively. Different molecules have been engineered for this specific purpose in the last few years and are currently under investigation. Some have passed phases I and II clinical trials showing promising effects on glucose control and weight loss (20, 26–30).

**Figure 1:**
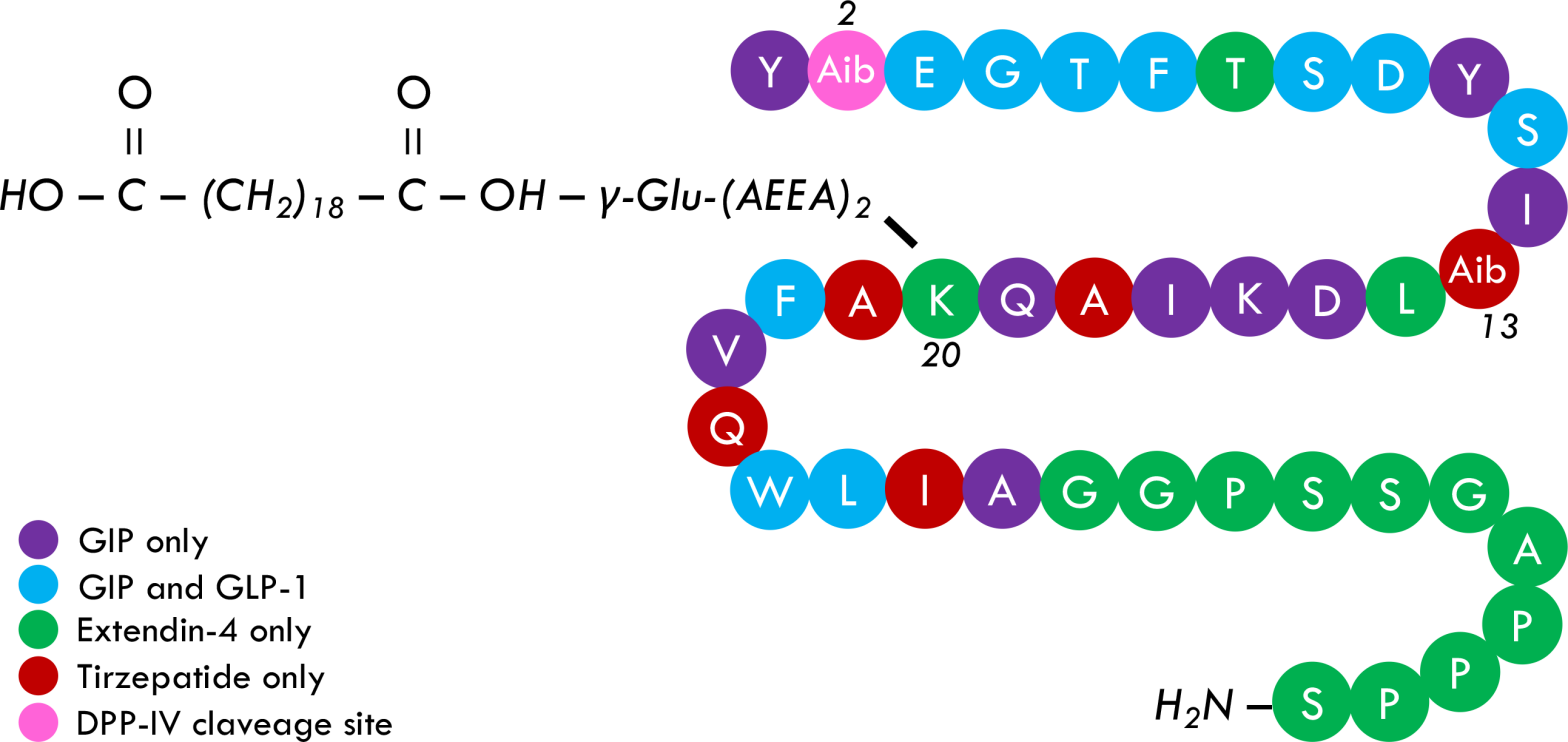
Tirzepatide primary structure. TZP is a 39-peptide containing different native and not-native incretins fragments, including GIP, GIP, GLP-1, and Extendin-4 (and glucagon, not shared). Alpha aminobutyric acid is placed in positions 2 and 13 and provides TZP an intrinsic resistance to DPP-IV attack and more structural stability, respectively. A 20-carbon fatty acid, namely eicosanedioic acid, is linked to Glu. A 2xAdo unit is attached to the lysine residue in position 20 and allows TZP to be bound to albumin, consequently increasing its half-life to five days. It is currently debated whether the acylation may also increase receptor bounding stability, hypothetically affecting the pharmacological potency of TZP.

Tirzepatide (LY3298176) successfully passed early phases of clinical investigation and showed relevant effects on glycemic control and body weight in phase III, randomized clinical trials (RCTs). Tirzepatide (TZP) is a 48 KDa synthetic peptide composed of 39 amino acids. The basic structure of TZP (**Figure 1**) comprises several highly affine fragments of other incretins, including GIP, GIP and GLP-1, Extendin-4, and glucagon. Moreover, two residues of alpha aminobutyric acid are placed in positions 2 and 13 with the strategic purposes to strengthen TZP resistance to DPP-IV cleavage and ameliorate molecular stability, respectively. A 20-carbon fatty acid linked to the lysine residue in position 20 of the primary structure allows TZP to be bound to circulating albumin. Acylation technology, previously adopted and developed for Liraglutide and Semaglutide, increases TZP half-life to around five days, making it administrable once weekly (28, 31–33).

TZP is an unbalanced dual agonist of GIPR and GLP-1R. Preclinical trials demonstrated that TZP (LY3298176) exhibited for GIPR the same affinity as native GIP and a five-fold weaker affinity for GLP-1R than the native GLP-1 (20). The potency of TZP for GIPR is similar to GIP and thirteen-fold lower for GLP-1R than GLP-1 (20). The potency of TZP for GLP-1R was also inferior compared to Semaglutide (20). In *in vitro* studies, TZP stimulated ß-cells response more efficiently than observed with GIP and GLP-1 alone. Also, *in vivo*, TZP enhanced insulin secretion by acting on both GIPR and GLP-1R (20).

Efficacy and safety of TZP were investigated in phase III, RCTs of the SURPASS program to verify its efficacy and safety in improving glycemic control in different therapeutic lines (**Table 1**). TZP monotherapy versus placebo was investigated in the SURPASS-1 (34). A head-to-head comparison between TZP and Semaglutide as an add-on to metformin in uncontrolled T2D was analyzed in the SURPASS-2 (35). The SURPASS-3 and SURPASS-4 examined the safety and efficacy of TZP as an intensification of previous oral treatment (metformin and sodium-glucose transporter 2 inhibitors in the SURPASS-3; metformin, sulfonylureas, or sodium-glucose transporter 2 inhibitors alone or in variable combinations in the SURPASS-4) versus insulin Degludec and Glargine respectively (36, 37). The latter trial was carried out in a population at high cardiovascular risk. TZP was administered subcutaneously in three doses (5, 10, and 15 mg once a week). Overall, TZP induced a relevant reduction in HbA_1c_ with superiority versus direct comparators, and a relevant percentage of patients achieved optimal glucose control (i.e., HbA_1c_ <7% or ≤6.5%) or reverted to euglycemia (HbA_1c_ <5.7%). Also, weight loss was impressive at all tested doses.

**Table 1:** Summary of the SURPASS program, phase III clinical trials (34–37).

Study(year)	Study duration(weeks)	Intervention vs. comparator	Randomization(number of participants)	Baseline characteristics	Main findings
*SURPASS-1* **Rosenstock J (2021)**	40	Tirzepatide vs. placebo (monotherapy in naïve T2D patients)	1:1:1:1 Tirzepatide 5 mg qw (125) Tirzepatide 10 mg qw (125) Tirzepatide 15 mg qw (125) Placebo qw (121)	-HbA_1c_ 7.9% -54y -women 48% -mean diabetes evolution 4.7y -BMI 31.9 Kg/m^2^	HbA_1c_ -1.87% Tirzepatide 5 mg -1.89% Tirzepatide 10 mg -2.07% Tirzepatide 15 mg +0.04 placebo -HbA_1c <_7% 87-92% (Tirzepatide) vs. 20% (placebo) -HbA_1c ≤_6.5% 81-86% (Tirzepatide) vs. 10% (placebo) - HbA_1c <_5.7% 31-52% (Tirzepatide) vs. 1% (placebo) Weight loss -7 to -9.5 kg (Tirzepatide 5 to 15 mg)
*SURPASS-2* **Frías JP (2021)**	40	Tirzepatide vs. Semaglutide (T2D patients with poor glycemic control while on metformin)	1:1:1:1 Tirzepatide 5 mg qw (470) Tirzepatide 10 mg qw (469) Tirzepatide 15 mg qw (470) Semaglutide 1 mg qw (469)	-HbA_1c_ 8.28% -56.6y -women 53% -mean diabetes evolution 8.6y -BMI 31.9 kg/m^2^ -waist 109.3 cm -eGFR 96 ml/min	HbA_1c_ -2.01% Tirzepatide 5 mg -2.24% Tirzepatide 10 mg -2.3% Tirzepatide 15 mg -1.86% Semaglutide 1mg -HbA_1c <_7% 82-86% (Tirzepatide) vs. 79% (Semaglutide) -HbA_1c ≤_6.5% 69-80% (Tirzepatide) vs. 64% (Semaglutide) - HbA_1c <_5.7% 27-46% (Tirzepatide) vs. 19% (Semaglutide) Weight loss -7.6 to -11.2 Kg (Tirzepatide 5-15 mg) vs. -5.7 Kg (Semaglutide 1 mg)
*SUPRASS-3* **Ludvik B (2021)**	52	Tirzepatide vs. insulin Degludec (as an add-on to metformin +/- sodium glucose transporter 2 inhibitors)	1:1:1:1 Tirzepatide 5 mg qw (361) Tirzepatide 10 mg qw (361) Tirzepatide 15 mg qw (361) titrated insulin Degludec (361)	-HbA_1c_ 8.17% -18y -diabetes duration 8.4y -BMI >25 kg/m^2^	HbA_1c_ -1.9% Tirzepatide 5 mg, qw -2.2% Tirzepatide 10 mg, qw -2.37% Tirzepatide 15 mg, qw -1.34 titrated insulin Degludec -HbA_1c <_7% 82-93% (Tirzepatide) vs. 61% (titrated insulin Degludec) Weight loss -7.5 to -11.2 Kg (Tirzepatide 5-15 mg) vs. +2.3 Kg (titrated insulin Degludec)
*SURPASS-4* **Del Prato S (2021)**	52	Tirzepatide vs. Insulin Glargine (as an add-on to metformin +/- secretagogues +/- sodium glucose transporter 2 inhibitors in any combinations in patients at high cardiovascular risk)	1:1:1:3 Tirzepatide 5 mg qw (329) Tirzepatide 10 mg qw (328) Tirzepatide 15 mg qw (338) titrated insulin Glargine (1000)	-HbA_1c_ 8.5% ->18y -diabetes evolution 11.8y -BMI >25 kg/m^2^	HbA_1c_ -2.43% Tirzepatide 10 mg, qw -2.28% Tirzepatide 15 mg, qw -1.44% titrated insulin Glargine MACE-4 events (cardiovascular death, myocardial infarction, stroke, hospitalization for unstable angina): Tirzepatide compared to insulin Glargine hazard ratio 0.74 (95% CI 0.51–1.08).

## Comment on the SURPASS-5 trial

The results of the 40-week double-blind randomized, placebo-controlled, phase III clinical trial SURPASS-5 (NCT04039503) have recently been published (38). This was a multicentric study involving 45 centers involved in diabetes clinical research from eight different countries (Czech Republic, Germany, Japan, Poland, Puerto Rico, Slovak Republic, Spain, and the USA) and assessing the efficacy and safety of TZP compared to placebo as add-on to insulin Glargine with or without metformin in uncontrolled T2D individuals.

The primary outcome was to test TZP 10 and 15 mg once weekly for superiority versus placebo in reducing HbA_1c_ from baseline to 40 weeks. The secondary outcome included superiority of TZP 5 mg qw versus placebo in reducing HbA_1c_ from baseline to 40 weeks. Additional outcomes were: superiority of TZP 5, 10, and 15 mg qw versus placebo in terms of weight loss; percentage of patients who would have achieved optimal glycemic control (HbA_1c <_7%); reduction of fasting blood glucose values, reduction of daily mean blood glucose assessed by 7-point glucose monitoring (fasting, pre-meal, post-meal and bedtime blood glucose); percentage of patients who would have achieved weight loss ≥5%; percentage of change in insulin Glargine dose; the number of severe hypoglycemia events (<54 mg/dL); percentage of patients who would have achieved blood glucose normalization (HbA_1c <_5.7%).

Patients were screened for eligibility and recruited based upon the following key elements: had received a diagnosis of T2D according to diagnostic standard by the World Health Organization; HbA_1c_ 7-10.5%; body mass index >23 kg/m^2^ with irrelevant variation ( ± 5%) in last three months; stable (at least three months before) daily dose of insulin Glargine (>20 IU/day) plus daily metformin ≥ 1.500 g; age ≥18 years.

Exclusion criteria included: type 1 diabetes, either acute or chronic pancreatitis, proliferative diabetic retinopathy or macular diabetes, non-proliferative diabetic retinopathy requiring urgent treatment, either acute or chronic hepatopathy with clinic or laboratory evidence of liver injury (e.g., 3-fold elevation in plasmatic transaminases), severe hypoglycemia or unawareness hypoglycemia, gastroparesis, previous bariatric surgery, concomitant use of certain medications delaying gastric emptying, established coronary disease or severe chronic heart failure (NYHA class III or IV), glomerular filtration rate <30 mL/min/1.73 m^2^ in patients not taking metformin and glomerular filtration rate <45 mL/min/1.73 m^2^ in those on metformin, adrenal insufficiency or severe thyroid dysfunction, positive family history of medullary thyroid cancer or personal basal serum calcitonin ≥35 ng/L, active malignancy over the last five years, transplant recipients, alcoholism, and psychiatric disorders.

Four hundred seventy-five patients (44% women) were randomized, with a median age of 61 years and an average HbA_1c_ of 8.3%. Among them, 95% concluded the trial. Patients were allocated in four groups and randomized (1:1:1:1) to receive TZP 5 mg (n: 166), TZP 10 mg (n: 119), TZP 15 mg (n:120), or placebo (n: 120).

TZP was started at 2.5 mg qw and up-titrated every four weeks until achieving the final dose. Insulin Glargine was administered once a day at bedtime and titrated every three days to maintain fasting glucose between 71 and 100 mg/dL. Insulin doses were increased by two, four, six, or eight units in case of fasting glycemia between 101 – 119, 120 – 139, 140 – 179, and more than 180 mg/dL, respectively. Insulin therapy was steadied within four weeks after the randomization (stabilization period) and then was managed with a treat-to-target purpose for the remaining 36 weeks.

After 40 weeks, HbA_1c_ reduction was superior in the group treated with Tirzepatide 5 mg (-2.1%), 10 mg (-2.4%), and 15 mg (-2.3%) compared to placebo (-0.9%) with an estimated treatment difference of -1.24% in favor of TZP 5 mg, -1.53% in favor of TZP 10 mg, and -1.47% in favor of Tirzepatide 15 mg (p <.001 for superiority over placebo).

Around 85-90% of participants randomized to receive TZP achieved HbA_1c <_7%. This percentage was significantly higher compared to the placebo (34%). A normalization in glucose levels (HbA_1c <_5.7%) was obtained in 26-62% of patients on TZP (5-15 mg), with a relevant difference compared to placebo (2.5%).

A considerable body weight loss was reached after 40 weeks of treatment with TZP consisting of -5.4 kg (5 mg), -7.5 kg (10 mg), and -8.8 kg (15 mg). Patients randomized to placebo obtained a slight but relevant weight gain compared to baseline (+1.6 kg); therefore, the estimated treatment difference between TZP and placebo was -7 Kg (TZP 5 mg), -10 kg (TZP 10 mg), -8.8 Kg (TZP15 mg, all p <.001). Overall, a more significant proportion of participants (54-84%) randomized to TZP (5-15 mg) achieved a clinically relevant body weight loss (≥5%) compared to placebo (6%).

Fasting glucose levels decreased more evidently with TZP (-61 to -68 mg/dL) than placebo (-39 mg/dL). Similarly, average of daily glucose levels was lower in TZP (-67 to -74 mg/dL) than placebo (-39 mg/dL).

The daily dose of insulin Glargine increased by 75% from baseline to 40 weeks in the placebo group while increasing by 13% and 8% with TZP 5 and 10 mg, respectively. Patients randomized to TZP 15 mg exhibited a slight reduction in daily Glargine dose requirement by 11%.

Hypoglycemia was defined as glucose level <54 mg/dL. The hypoglycemic risk was low in all groups, and it did not change up to 44 weeks (including a 4-week study extension analysis aimed to assess safety end-points specifically). The incidence of hypoglycemia was 14-19% in TZP arms and 13% in the placebo group. Only three episodes of level 3 hypoglycemia were detected (two in the group TZP 10 mg and one in TPZ 15 mg).

Twenty-four patients withdrew from the study: seven of them assumed TZP 5 mg (three for adverse events and four because of personal decision); four were on TZP 10 mg (one due to protocol violation and three because of personal decision), ten were on Tirzepatide 15 mg (two for adverse events, one for missed follow-up, two because of protocol violation, and five because of personal decision). Safety monitoring was recorded for up to 17 months; the most common adverse events were nausea (13-18% with TZP vs. 3% with placebo), diarrhea (12-21% on TZP vs. 2% on placebo), loss of appetite (7-14% on TZP vs. 2% on placebo), vomiting (7-12% on TZP vs. 2% on placebo). Serious adverse events were uncommon, and no between-group differences were found.

## Basal insulin intensification with GLP-1RA or dual GIP and GLP-1RA

Basal insulin intensification is necessary for managing specific situations such as T2D with long duration and poor glucometabolic control suggestive of reduced pancreatic reserve (39). A timely basal insulin prescription, when appropriate, is recommended to improve glucose control rapidly, reduce glucotoxicity, and ameliorate beta-cell reserve over time, facilitating further pharmacologic strategies to reach more appropriate therapeutic goals (40). Nonetheless, it is well-known that only a minority of insulin-treated patients have a further chance to achieve a tailored glycemic control (41) due to delayed or inadequate basal insulin titration over time or deferred therapeutic intensification (42).

GLP-1RAs added to basal insulin are demonstrated to improve glucometabolic control without increasing hypoglycemic risk and prevent weight gain compared to a further titration of basal insulin in T2D patients failing to reach adequate glycemic control (43–46). The mechanisms leading to favorable outcomes when adding a GLP-1RA to basal insulin include improving pancreatic reserve, ameliorating insulin sensibility, suppressing glucagon secretion, weight loss, cardiovascular and microvascular protection, and increasing treatment adherence, especially when considering once-weekly GLP-1RAs. In the SURPASS-5, TZP confirmed the importance of injectable incretin-based therapy in this cluster of patients and appeared to be a further therapeutic tool for T2D.

**Figure 2:**
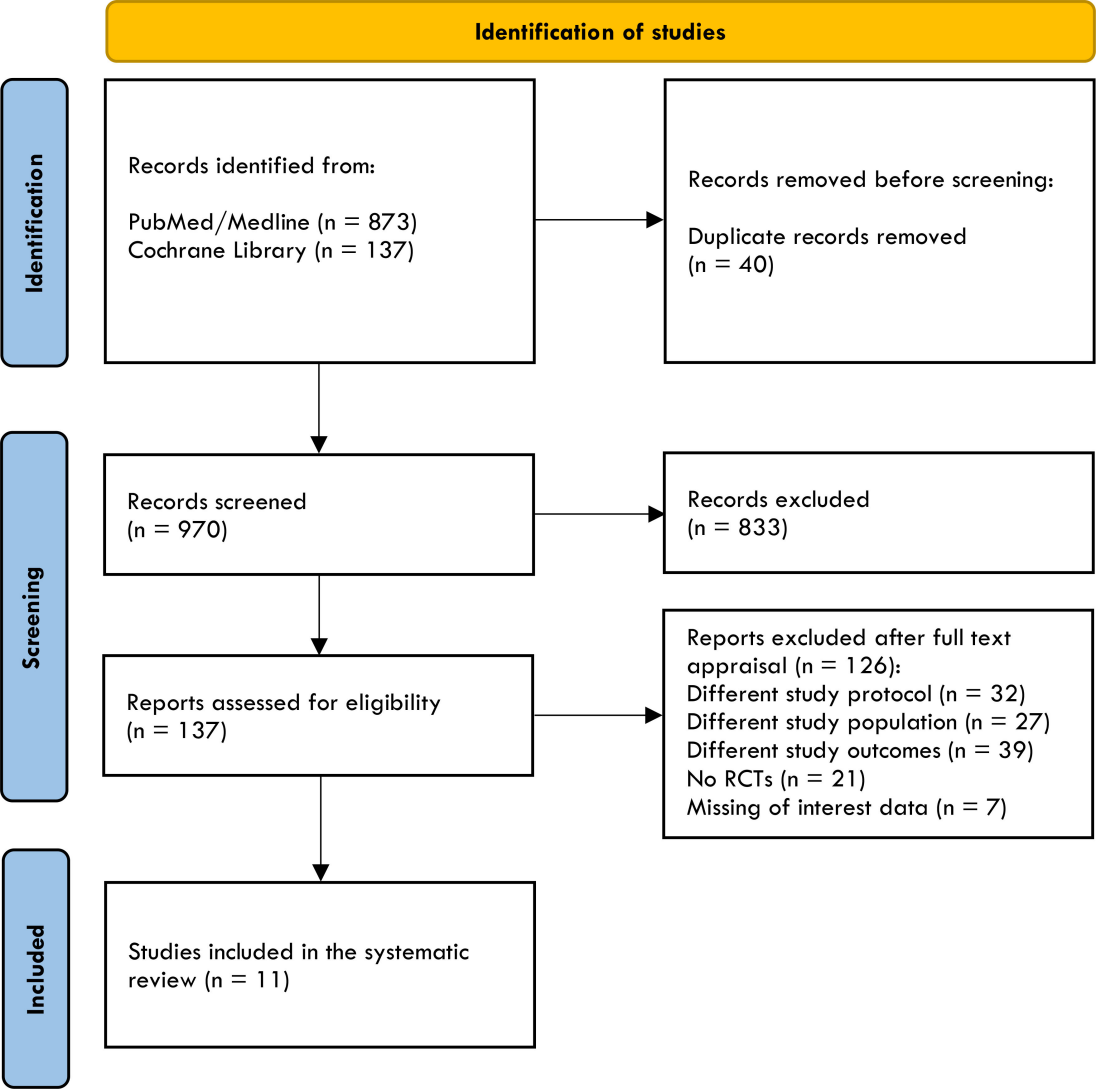
The PRISMA flow diagram of included studies.

In light of this evidence, we carry out a rapid review and meta-analysis to review and summarize the effect of injectable incretin-based therapy compared to placebo when added to basal insulin therapy in uncontrolled T2D. PubMed/MEDLINE, Cochrane Library were searched by GL and ADT using the following strategy: “(exenatide) or (liraglutide) or (lixisenatide) or (dulaglutide) or (albiglutide) or (semaglutide) or (tirzepatide) or (glp-1ra) or (“glucagon-like peptide 1 receptor agonist”) and (glargine) or (glargine) or (degludec) or (basal insulin)”. Database search was performed until 15 February 2022, and the timeline of extracted data ranged from 01 January 2011 to 15 February 2022. After duplicate removals, articles were screened and extracted for the synthesis according to a hierarchical strategy that included title, abstract, and full-text appraisal. Studies were screened and included based upon the following criteria: population with established diagnosis of T2D; age ≥18 years; basal insulin treatment at stable dose ( ± 20%) for at least six weeks before screening visits (100% of participants) with or without oral antihyperglycemic agents such as metformin, sulfonylureas, pioglitazone, DPP-IV inhibitors, sodium glucose transporter type 2 inhibitors; inadequate glucose control (HbA_1c_ ≥7%); clinically relevant follow-up period (≥24 weeks); intensification with a GLP-1RA (both short- and long-acting) or dual GIP/GLP-1RA specifically designed for the treatment of T2D (i.e. Lixisenatide 10-20 μg/day; Exenatide 5-10 μg/bid; Liraglutide up to 1.8 mg/day; Dulaglutide 1.5 mg/qw; Exenatide 2 mg/qw; Semaglutide 0.5 e 1 mg/qw; Tirzepatide 5, 10, 15 mg/qw) as an add-on to basal insulin and not vice versa; placebo as comparator group; primary and secondary outcomes including mean change from baseline to study end in HbA_1c_, fasting glycemia, body weight, insulin dose, number of individuals experiencing symptomatic hypoglycemia or glucose levels (<70 mg/dL); number of patients experiencing adverse events. Fixed-ratio formulations and separately administered basal insulin plus GPL-1RA were both included. The search was further restricted to RCTs written in English. Eleven RCTs have been included in the meta-analysis (38, 43–52) as show in **Table 2**. Studies focusing on specific ethnic groups were excluded (53, 54). Other trials exploring differences between GLP-1RAs added to basal insulins (free or fixed-ratio) and basal insulins plus placebo were excluded as participants were insulin naïve at baseline (55–57). One trial was excluded because Liraglutide 3 mg is formally approved for obesity (58). The identification of RCTs included in the systematic review is shown in **Figure 2**.

**Table 2:** Summary of RCTs included in the systematic review.

Study (reference)NCT(year)	Type of study	Baseline characteristics	Intervention vs. comparator	Concomitant antihyperglycemic agents	Run-in period	Duration(weeks)	Main outcomes
Buse et al. (43) NCT00765817 (2011)	Individually randomized parallel-group, double-blind	T2D; HbA_1c_ 8.3%; FPG 146 mg/dL; body weight 94.5 kg; BMI 33 kg/m^2^; insulin dose 48 IU/day (Glargine); mean age 59y; diabetes evolution 12y	Exenatide 10 μg x twice a day vs. placebo	+/- Metformin or Pioglitazone	–	4 (Exenatide 5 μg x 2/day) + 26 (Exenatide 10 μg x 2/day)	Change from baseline in HbA_1c_, fasting plasma glucose, 7-point self-monitored glucose, lipid profile, body weight, waist circumference, diastolic and systolic arterial pressure, daily insulin dose, percentage of participants achieving HbA_1c <_7% and ≤6.5%, number of patients experiencing symptomatic or severe hypoglycemia.
GetGoal-Duo1 (44) NCT00975286 (2013)	Individually randomized parallel-group, double-blind	T2D (>1 year); HbA_1c_ (before randomization) 7.6%; FPG (before randomization) 120 mg/dL; body weight 87 kg; BMI 31.8 kg/m^2^; insulin dose 44 IU/day (Glargine); mean age 56y; diabetes evolution 9y	Lixisenatide 20 μg/day vs. placebo	Metformin ≥1,500 mg/day +/- Sulfonylureas +/- Pioglitazone	Twelve weeks to start and titrate insulin Glargine	24	Change from baseline in HbA_1c_, fasting plasma glucose, 7-point self-monitored glucose, 2-h postprandial glucose, glycemic excursion, body weight, waist circumference, daily insulin dose, percentage of participants achieving HbA_1c <_7% or ≤6.5%, percentage of patients reaching weight reduction ≥5%, number of patients experiencing symptomatic or severe hypoglycemia, percentage of patients requiring rescue therapy (glycemia >200 mg/dl, HbA_1c >_9%, week 0 – 8; glycemia >180 mg/dl, HbA_1c >_8.5%, week 8 – 24).
GetGoal-L (45) NCT00715624 (2013)	Individually randomized parallel-group, double-blind	T2D (>1 year); HbA_1c_ 8.4%; FPG 145 mg/dL; body weight 88.7 kg; BMI 32 kg/m^2^ insulin dose 55 IU/day (50% Glargine) insulin use 3y; mean age 57y; diabetes duration 12.5y	Lixisenatide 20 μg/day vs. placebo	Metformin ≥1,500 mg/day	–	24	Change from baseline in HbA_1c_, fasting plasma glucose, 7-point self-monitored glucose, 2-h postprandial glucose, glycemic excursion, body weight, daily insulin dose, percentage of participants achieving HbA_1c <_7% or ≤6.5%, percentage of patients reaching body weight reduction ≥5%, number of patients experiencing symptomatic or severe hypoglycemia, treatment satisfaction score, percentage of patients requiring rescue therapy (glycemia >200 mg/dl, HbA_1c >_9%, week 0 – 8; glycemia >180 mg/dl, HbA_1c >_8.5%, week 8 – 24).
DUAL II (46) NCT01392573 (2014)	Individually randomized parallel-group, double-blind	T2D; HbA_1c_ 8.8%; FPG 174 mg/dL; body weight 94.5 kg; BMI 33.7 kg/m^2^; insulin dose 29 IU/day (mostly Glargine); mean age 58y; diabetes duration 10.5y	Liraglutide (mean final dose of 1.62 mg/day) in fixed-combination with insulin Degludec vs. insulin Degludec alone	Metformin ≥1,500 mg/day +/- Sulfonylureas or glinides	–	26	Change from baseline in HbA_1c_, fasting plasma glucose, 9-point self-monitored glucose, body weight, 2-h postprandial glucose, percentage of participants achieving HbA_1c <_7% and ≤6.5%, percentage of participants achieving HbA_1c <_7% with or without confirmed hypoglycemia or weight gain, changes in laboratory-measured FPG, 9-point plasma glucose (PG) profiles, and body weight. percentage of patients experiencing hypoglycemia, number of documented symptomatic hypoglycemic events, percentage of participants with severe symptomatic hypoglycemia
Ahmann et al. (47) NCT01617434 (2015)	Individually randomized parallel-group, double-blind	T2D (>1 year); HbA_1c_ 8.2%; FPG 148 mg/dL; body weight 91 kg; BMI 32 kg/m^2^; insulin dose 40 IU/day (any basal insulin); mean age 58y; diabetes duration 12y	Liraglutide 1.8 mg/day vs. placebo	Metformin ≥1,500 mg/day	–	26	Change from baseline in HbA_1c_, fasting plasma glucose, 7-point self-monitored glucose, body weight, percentage of participants achieving HbA_1c <_7% or ≤6.5%, number of patients experiencing mild and severe hypoglycemic episodes, number of adverse events.
LixiLan-L (48) NCT02058160 (2016)	Individually randomized parallel-group, open-label	T2D (>1 year); HbA_1c_ (before randomization) 8.1%; FPG (before randomization) 132 mg/dL; body weight 87.7 kg; BMI 31 kg/m^2^; Insulin Glargine dose 35 IU/day; insulin use 3y; mean age 58y; diabetes evolution 12y	Lixisenatide (mean final dose of 17 μg/day) in fixed-combination with insulin Glargine vs. insulin Glargine alone	Metformin ≥1,500 mg/die +/- Sulfonylureas or sodium-glucose transported type 2 inhibitors or Pioglitazone or Dipeptidyl peptidase IV inhibitor	Six weeks to titrate insulin dose	30	Change from baseline in HbA_1c_, fasting plasma glucose, 7-point self-monitored glucose, body weight, 2-h postprandial glucose, percentage of participants achieving HbA_1c <_7% or ≤6.5%, percentage of participants achieving HbA_1c ≤_7% without a gain in body weight and no documented hypoglycemia, percentage of patients experiencing hypoglycemia, number of documented symptomatic hypoglycemic events, percentage of patients requiring rescue therapy, percentage of participants with severe symptomatic hypoglycemia
DUAL V (49) NCT01952145 (2016)	Individually randomized parallel-group, double-blind	T2D (>1 year); HbA_1c_ 8.3%; FPG 160 mg/dL; body weight 88 kg; BMI 31.7 kg/m^2^; insulin dose 31 IU/day mostly Glargine); mean age 58.5y; diabetes duration 11.6y;	Liraglutide (mean final dose of 1.48 mg/day) in fixed-combination with insulin Degludec vs. insulin Glargine alone	Metformina ≥1,500 mg/die	–	26	Change from baseline in HbA_1c_, body weight, and the number of confirmed hypoglycemic episodes.
AWARD-9 (50) NCT02152371 (2017)	Individually randomized parallel-group, double-blind	T2D; HbA_1c_ 8.4%; FSG 157 mg/dL; body weight 93 kg; BMI 32.7 kg/m^2^; insulin dose 38 IU/day (Glargine); Glargine use 2y; Mean age 60.5y; Diabetes evolution 12y	Dulaglutide 1.5 mg/qw vs. Placebo	+/- Metformin ≥1,500 mg/die	–	28	Change from baseline in HbA_1c_, fasting serum glucose, 7-point self-monitored serum glucose, body weight, daily mean insulin dose, percentage of participants with self-reported events of hypoglycemia, number of participants with investigator reported and adjudicated cardiovascular events, percentage of participants discontinuing the study due to severe, persistent hyperglycemia, number of participants with thyroid neoplasms, number of participants with Dulaglutide anti-drug antibodies, percentage of participants achieving HbA_1c_ targets of <7.0% or ≤6.5%, percentage of participants achieving HbA_1c_ target of <7.0% and without weight gain or documented symptomatic hypoglycemic episodes, rate of hypoglycemic events
SUSTAIN 5 (51) NCT02305381 (2018)	Individually randomized parallel-group, double-blind	T2D; HbA_1c_ 8.4%; FPG 156 mg/dL; body weight 92 kg; BMI 32.8 kg/m^2^; insulin dose 39 IU/day (mostly Glargine); mean age 59y; diabetes duration 13y	Semaglutide 0.5 and 1 mg/qw vs. Placebo	+/- Metformina	–	30	Change from baseline in HbA_1c_, body weight, fasting plasma glucose, daily insulin dose, systolic and diastolic blood pressure, patient-reported outcomes, diabetes treatment satisfaction questionnaire, percentage of participants achieving HbA_1c_ targets of <7% or ≤6.5%
DURATION-7 (52) NCT02229383 (2018)	Individually randomized parallel-group, double-blind	T2D; HbA_1c_ 8.5%; FPG 147 mg/dL; body weight 94 kg; BMI 34 kg/m^2^; insulin dose 51 IU/day; Mean age 58y; Diabetes evolution 11y	Exenatide 2 mg/qw vs. placebo	+/- Metformin	Eight weeks to titrate insulin dose	28 + 10 (supplemental follow up)	Change from baseline in: HbA_1c_, body weight, 2-hour postprandial glucose after a standard meal tolerance test, daily insulin dose, systolic blood pressure, percentage of participants achieving HbA_1c_ targets of <7% without weight gain and major hypoglycemia
SURPASS-5 (38) NCT04039503 (2022)	Individually randomized parallel-group, double-blind	T2D; HbA_1c_ 8.3%; FSG 161 mg/dL; body weight 95.5 kg; BMI 33.4 kg/m^2^; insulin dose (Glargine) 34 IU/day; mean age 61y; diabetes duration 13y	Tirzepatide 5, 10 e 15 mg/qw vs. Placebo	+/- Metformin	–	40	Change from baseline in: HbA_1c_, body weight, fasting serum glucose, daily average 7-Point self-monitored blood glucose, weight reduction ≥5%, daily insulin dose, percentage of participants achieving HbA_1c_ targets of <7%, ≤6.5%, and ≤5.7%, rate of severe hypoglycemia

T2D, type 2 diabetes; HbA_1c_, glycated hemoglobin; FPG, fasting plasma glucose; FSG, fasting serum glucose; BMI, body mass index; IU, international units.

**Figure 3:**
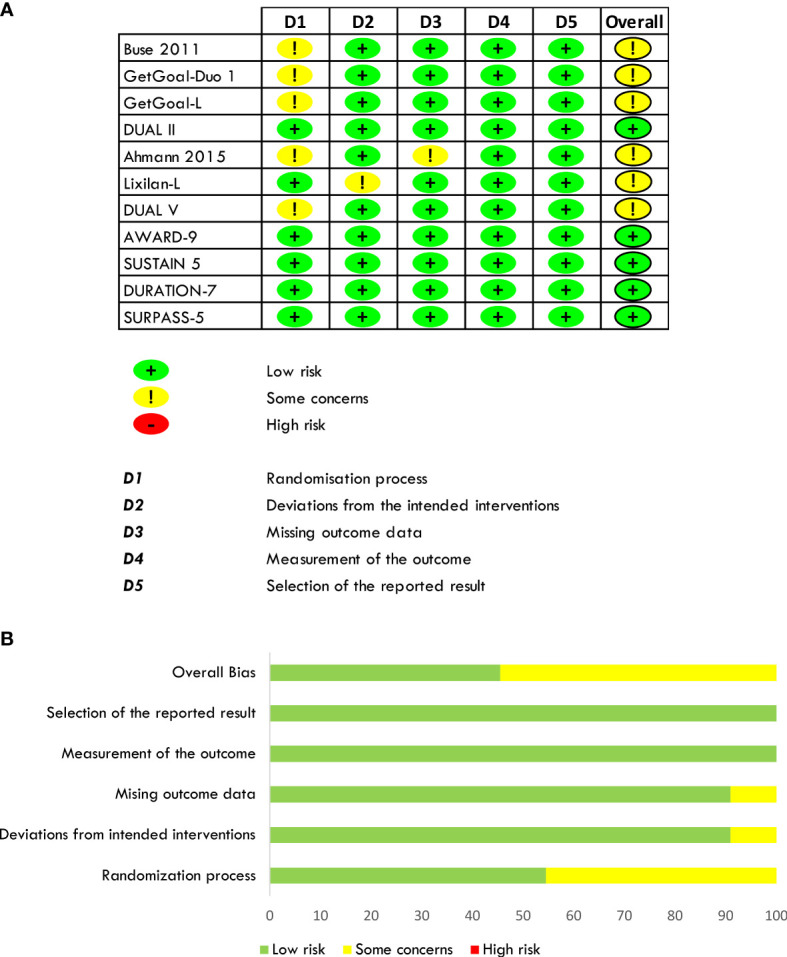
Quantification of the risk of bias of included studies **(A, B)**.

The risk of bias was estimated by RoB2 for individual randomized, parallel-group trials (59). Six out of eleven trials have been considered at moderate risk of bias (some concerns) due to the randomization process for different baseline characteristics. In Buse et al., participants randomized to receive the placebo compared to those who received Exenatide had higher baseline HbA_1c_ and fasting plasma glucose, lower body weight, and baseline insulin dose, and were predominantly men than women with a higher percentage of metformin users. In the GetGoal-Duo 1 trial, participants randomized to receive insulin Glargine had longer diabetes evolution than those assuming insulin Glargine/Lixisenatide, and this difference might have been responsible for different beta-cell reserve between the two study groups. In the GetGoal-L trial, participants randomized to placebo had higher insulin doses and higher body weight, while in Ahmann et al., patients on placebo were younger, more men than women, and had higher body weight. Moreover, a possible attrition bias might not be excluded because a significant percentage of patients withdrew in both groups. In the DUAL V, participants randomized to Liraglutide had higher body weight and HbA_1c_ and were more hypertensive. All trials were double-blind except for the Lixilan-L, designed as open-label, but analyses were carried out blind. All trials were sponsored by the pharmaceutical industry (Eli Lilly and Company and Amylin Pharmaceuticals; Sanofi; Sanofi; NovoNordisk; NovoNordisk; Sanofi; NovoNordisk; Eli Lilly and Company; NovoNordisk; AstraZeneca; Eli Lilly and Company). The risk of bias in the included studies is shown in **Figures 3A, B**.

All analyses were performed by RevMan 5.4.1 with a random-effect model, considering a p-value <.05 as statistically significant. Four outcomes (change in HbA_1c_, fasting plasma glucose, body weight, and basal insulin dose from baseline to study end) were analyzed as continuous variables, and results were summarized in weighted mean difference or standardized mean difference (change in basal insulin dose that was assessed by different scales). One outcome (percentage of participants achieving HbA_1c_<7%) was analyzed as a dichotomous variable, and results were synthesized in terms of risk ratio (RR). Heterogeneity was assessed by I^2^. The level of heterogeneity was considered substantial in the case of I^2^>50%. Standard error, 95% confidence interval (CI), and interquartile range were used alternatively to estimate the standard deviation when missed.

The metanalysis includes 5,323 patients (56-61 years) with T2D inadequately controlled (HbA_1c_ 7-10.5%), baseline BMI 23-45 kg/m^2^, mean diabetes duration of 9-12 years, and randomized to receive a GLP-1RA or GIP/GLP-1RA versus placebo in add on to basal insulin for at least 24 weeks (24-40 weeks).

**Figure 4:**
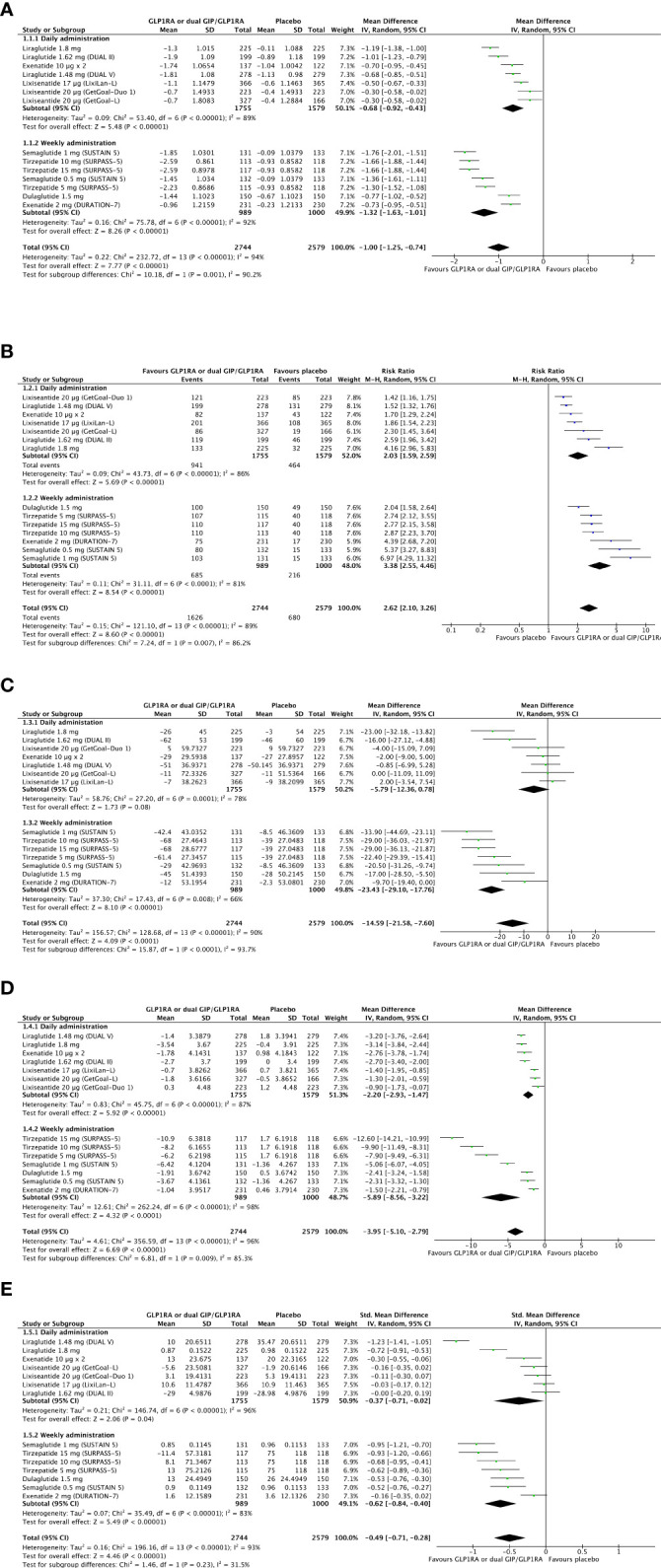
Forest plots of meta-analysis for change in HbA_1c_ **(A)**, chance of achieving optimal glucose control as HbA_1c_ <7% **(B)**, and change in fasting plasma glucose **(C)** from baseline to the last available follow-up (intention-to-treat analyses). Forest plot of meta-analysis for change in body weight **(D)** from baseline to the last available follow-up (intention-to-treat analysis). Forest plot of meta-analysis for change in the mean daily dose of basal insulin **(E)** from baseline to the last available follow-up (intention-to-treat analysis).

The weighted overage baseline HbA_1c_ of included studies was 8.3% before the randomization. Combining a GLP-1RA or dual GIP/GLP-1RA to basal insulin provides a significant improvement in glucose control (Δ HbA_1c_ = -1%, 95% CI -1.25; -0.74, I^2^ 94%) compared to progressive basal insulin titration with a rigorous intention-to-treat approach. Also, the probability of reaching optimal glucose control (HbA_1c <_7%) is significantly higher with GLP-1RA or dual GIPR/GLP-1R added to basal insulin as compared to progressive basal insulin titration with a rigorous intention-to-treat approach (RR 2.62, 95% CI 2.10; 3.26, I^2^ 89%). Baseline weighted overage fasting plasma glucose (FPG) was 149.6 mg/dL. Overall reduction in FPG is significantly higher when GLP-1RAs or dual GIPR/GLP-1R is added to basal insulin (Δ FPG = -14.6 mg/dL, 95% CI -21.6; -7.6, I^2^ 90%). In this setting, daily administered GLP-1RAs added to basal insulin provides a less relevant improvement in glycemic control (Δ HbA_1c_ = -0.68%, 95% CI -0.92; -0.43, I^2^ 89%/RR HbA_1c <_7% = 2.03, 95% CI 1.59; 2.59, I^2^ 86%/Δ FPG = -5.9 mg/dL, 95% CI -12.4; 0.8, I^2^ 78%) than weekly administered analogues, including Tirzepatide (Δ HbA_1c_ = -1.32%, 95% CI -1.63; -1.01, I^2^ 92%/RR HbA_1c <_7% = 3.38, 95% CI 2.55; 4.46, I^2^ 81%/Δ FPG = -23.4 mg/dL, 95% CI -29; -18, I^2^ 66%). Results are shown in **Figures 4A–C**.

The weighted average body weight was 91.3 kg at baseline. GLP-1RA or dual GIP/GLP-1RA added to basal insulin provides a relevant weight reduction compared to basal insulin titration with a rigorous intention-to-treat approach (Δ weight = -3.95 kg, 95% CI -5.1, -2.79, I^2^ 96%). The magnitude of this difference appears less evident with daily than weekly administered analogues (Δ weight = -2.2 kg, 95% CI -2.93, -1.47, I^2^ 87% versus Δ weight = -5.9 kg, 95% CI -8.56; -3.22, I^2^ 98%). Forest plot of meta-analysis for change in body weight from baseline to the last available follow-up is shown in **Figure 4D**.

The average weighted insulin dose was 41 IU/day. GLP-1RA or dual GIP/GLP-1RA added to basal insulin compared to basal insulin titrated with a rigorous intention-to-treat approach is moderately effective in preventing further increase in daily basal insulin dose from achieving treat-to-target glycemic goals (Δ daily insulin dose, SMD -0.49, 95% CI -0.71; -0.28, I^2^ 93%) without specific difference between daily and weekly administered agonists and with a moderate intergroup heterogeneity (I^2^ 31.5%). Forest plot of meta-analysis for change in basal insulin dose from baseline to the last available follow-up is shown in **Figure 4E**.

The overall hypoglycemic risk was unaffected by adding a GLP-1RA or TZP to basal insulin compared to basal insulin alone titrated with a rigorous treat-to-target approach (RR = 1.01, 95% CI 0.86; 1.18, I^2^ 67%).

Adverse events were also registered and reported in RCTs, particularly those more frequently documented (usually with prevalence ≥5%). Reported descriptions are heterogeneous but are commonly imputable to gastrointestinal adverse events such as nausea (RR 4.45, 95% CI 3.19; 6.21, I^2^ 49%), diarrhea (RR 1.96, 95% CI 1.58; 2.42, I^2^ 0%), and vomiting (RR 4.44, 95% CI 3.15; 6.24, I^2^ 3%) without specific differences between daily rather than weekly administered analogues. Data are shown in **Table 3** with more details.

**Table 3:** Summary of the most common reported adverse events displayed by molecules and doses.

Molecules (dose)	NCT (year)	Nausea (%)	Loss of appetite (%)	Diarrhea (%)	Vomiting (%)	Dyspepsia (%)	Constipation (%)
Exenatide (10 μg x 2/day) vs. Placebo	NCT00765817 (2011)	40.6 vs. 8.1	n.a.	18.1 vs. 8.1	18.1 vs. 4.7	n.a.	10.1 vs. 1.6
Lixisenatide (17 μg/day) vs. Placebo	NCT02058160 (2016)	10.4 vs. 0.55	n.a.	4.4 vs. 2.7	3.6 vs. 0.5	5.2 vs. 0.6	n.a.
Lixisenatide (20 μg/day) vs. Placebo	NCT00715624 (2013)	29.3 vs. 9.6	n.a.	11.2 Vs. 6	9.8 vs. 1.2	5.2 vs. 0.6	n.a.
Lixisenatide (20 μg/day) vs. Placebo	NCT00975286 (2013)	27.3 vs. 4.9	n.a.	6.7 vs. 3.1	9.4 vs. 1.35	n.a	n.a
Liraglutide (1.48 mg/day) vs. Placebo	NCT01952145 (2016)	9.4 vs. 1.1	n.a.	7.2% vs. 2.5%	5% vs. 1.8%	n.a.	n.a.
Liraglutide (1.62 mg/day) vs. Placebo	NCT01392573 (2014)	6.5 vs. 3.5	n.a.	6.5 vs. 3.5	n.a.	n.a.	n.a.
Liraglutide (1.8 mg/day) vs. Placebo	NCT01617434 (2015)	22.2 vs. 3.1	n.a.	10.7 vs. 4.9	8.9 vs. 0.9	7.1 vs. 0.9	n.a.
Dulaglutide (1.5 mg/qw) vs. Placebo	NCT02152371 (2017)	12 vs. 1.3	n.a.	11.3 vs. 4	6 vs. 0	6 vs. 0	n.a.
Exenatide (2 mg/qw) vs. Placebo	NCT02229383 (2018)	5.2 vs. 3.9	n.a.	4.7 vs. 3.5	0.4 vs. 1.3	2.2 vs. 0	0.9 vs. 1.7
Semaglutide (0.5 mg/qw) Semaglutide (1.0 mg/qw) vs. Placebo	NCT02305381 (2018)	11.4 vs. 16.8 vs. 4.5	n.a.	4.5 vs. 6.9 vs. 1.5	6.1 vs. 11.5 vs. 3.0	n.a.	n.a.
Tirzepatide (5 mg/qw) Tirzepatide (10 mg/qw) Tirzepatide (15 mg/qw) vs. Placebo	NCT04039503 (2022)	12.9 vs. 17.6 vs. 18.3 vs. 2.5	6.9 vs. 12.6 vs. 14.2 vs. 1.7	12.1 vs. 12.6 vs. 20.8 vs. 10	6.9 vs. 7.6 vs. 12.5 vs. 2.5	6.9 vs. 8.4 vs. 5 vs. 1.7	6 vs. 6.7 vs. 6.7 vs. 1.7

n.a., not available.

## Discussion

GLP-1RAs have changed the therapeutic paradigm of T2D as they significantly improve glucose control, also contributing to pleiotropic effects, especially at the cardiovascular level (60), in different clinical scenarios and therapeutic approaches. As some examples, GLP-1RAs are safer and slightly more effective in improving glucose control, significantly reducing body weight, and preventing hypoglycemia than basal insulin in poorly controlled T2D with oral antihyperglycemic agents (61). In addition, GLP-1RAs added to basal insulin, as both free and fixed-ratio, perform better than more composite insulin strategies, such as basal plus and basal-bolus regimens in uncontrolled T2D (62, 63).

Herein we examined the role of GLP-1RAs and the novel dual GIP/GLP-1RA TZP as an add-on to basal insulin therapy compared to basal insulin alone in improving glucose control. Three specific outcomes (change in HbA_1c_, FPG, and chance to achieve HbA_1c <_7% from baseline to study end) were included to estimate treatment differences in terms of glycemic control. Change in body weight was calculated, and safety data were also analyzed.

Intra and intergroup heterogeneities have characterized our results; therefore, some considerations and comments should be discussed to understand the findings better. First, six out of eleven RCTs had some concerns about the risk of randomization bias and some differences in terms of the baseline characteristics of the compared group. One RCT (Liraglutide 1.8 mg) was also at risk of attrition bias due to a significative and asymmetric withdrawal (placebo: 23%; liraglutide 16%). Second, study designs are formally similar, but there are some differences. In the GetGoal-Duo 1, insulin naïve patients started (with further titration) insulin Glargine during the twelve-week run-in period, and then they were randomized. Therefore, this study fell into inclusion criteria as patients received basal insulin during the last three months before randomization. However, as expected in insulin naïve, the response to insulin treatment was sudden and considerable; therefore, glucose control improved significantly during the run-in period (HbA_1c_ from 8.6% to 7.6%). It is well-known that baseline HbA_1c_ is positively related to the magnitude of HbA_1c_ reduction upon an antihyperglycemic treatment is started (64); hence, the efficacy of Lixisenatide compared to the placebo might have been lessened. On the other hand, diabetes duration was slightly shorter compared to other RCTs, and this variable could have fostered better therapeutic response in both groups despite background different diabetes duration. Third, titration and final dose of GLP-1RAs and TZP were different in the included studies. Dulaglutide and Exenatide qw did not require any titration, and they were started at 1.5 and 2 mg from the first administration with global exposure to the specific GLP-1RA total dose of 28 weeks in both the AWARD-9 and DURATION-7. Lixisenatide was started at 10 μg once daily for two weeks and then continued to 20 μg/day for 22 weeks in GetGoal-Duo 1 and GetGoal-L. Exenatide bid was administered at the dose of 5 μg x 2/day for four weeks, and then it was up-titrated to 10 μg x 2/die for 26 weeks. Liraglutide was started at 0.6 mg once daily and up-treated by 0.6 mg every week up to 1.8 mg/day (24 weeks). In the DUAL II, DUAL V and Lixilan-L, Liraglutide and Lixisenatide were titrated depending on basal insulin requirement for obtaining fasting glucose control on the bases of a treat-to-target approach; therefore, the final dose of Liraglutide and Lixisenatide was approximately 1.6 mg/day (DUAL II), 1.5 mg/day (DUAL V) and 17 μg/day (Lixilan-L). In the DUAL V, 40% of patients assuming Liraglutide/Degludec received 50 dose steps after 26 weeks corresponding to 1.8 mg of Liraglutide, with a wide range of variability also depending on baseline BMI (65). In the Lixilan-L, around 27% of participants received the final dose of 60 IU of Lixisenatide/Glargine, equivalent to the entire daily dose of Lixisenatide. Semaglutide was started at 0.25 mg once a week, then increased to 0.5 mg and 1 mg every four weeks. Therefore, in the SUSTAIN-5, the final dose of Semaglutide was reached after four weeks in the arm randomized to 0.5 mg/qw and after eight weeks in those randomized to 1 mg/qw with global exposure to the intention-to-treat pre-established dose of 26 weeks (0.5 mg) and 22 weeks (1 mg). TZP was started at 2.5 mg once a week and progressively titrated by 2.5 mg every four weeks; thus, the final dose for each arm (5, 10, and 15 mg) was respectively achieved after four, twelve, and twenty weeks from randomization with a considerable difference in time of exposure to TZP 5 mg (36 weeks), TZP 10 mg (28 weeks), TZP 15 mg (20 weeks).

Once these considerations are discussed, GLP-1RAs or GIP/GLP-1RA added to basal insulin effectively improve glucose control, induce weight loss, and prevent unnecessary basal insulin over-titration with a reasonable chance to reduce the daily dose of basal insulin while improving glucose control. Most importantly, improvement in glucose control and weight reduction are obtained without increasing the overall risk of hypoglycemia and the number of severe hypoglycemic events. Safety data confirm that GLP-1RAs and TZP increase the risk of gastrointestinal adverse events (mostly mild-to-moderate), including nausea, diarrhea, and vomiting that are usually well-tolerated and resolved in a few weeks of treatment because of tachyphylaxis (especially with long-acting analogues and TZP). Weekly administered agonists performed better compared to daily ones. However, among daily administered analogues, Liraglutide appears to perform well, especially when administered separately in add-on to insulin Glargine (47). After excluding Liraglutide from the dataset, the levels of heterogeneity have been reduced moderately regarding the chance of reaching HbA_1c <_7% (I^2^ 42%) and considerably for change in fasting plasma glucose (I^2^ 0%), suggesting that Liraglutide compared to other daily administered GLP-1RAs is more effective in reducing those outcomes (data not shown). Short-acting GLP-1RAs exhibit a considerable fluctuation in circulating levels during the daytime that, in turn, produce an intermittent activation of GLP-1R, resulting in different pharmacodynamic effects compared to long-acting GLP-1RA (66). As a result, short-acting compared to long-acting GLP-1RAs are less effective in reducing fasting glucose and improving overall glucose control but are more effective in delaying gastric emptying and reducing glycemic excursion after meal ingestion (67). On the other hand, Liraglutide, Dulaglutide, and Exenatide LAR display similar efficacy in different therapeutic approaches, performing better than short-acting GLP-1RAs (68). The results of this metanalysis are in line with a previous one, thus confirming that therapeutic intensification with GLP-1RA is an effective and safe approach, especially using long-acting GLP-1RAs, mostly weekly than daily administered ones (69).

TZP looks like a further tool to improve glucose control as an add-on to basal insulin in this scenario. In addition to a potent antihyperglycemic effect, TZP leads to an impressive weight loss exceeding the results provided by other agents. When excluding TZP from the dataset, the overall efficacy of GLP-1RAs was reduced significantly and resulted in a slight body weight loss (-2.40 kg, 95% CI -3.04; -1.76) without subgroup differences (I^2^ 0%) between daily and weekly administered analogues (data not shown).

Overweight and obesity are most commonly observed in T2D, and this unhealthy relationship is expected to rise over time with growing detrimental consequences (70). So far, body weight management should be addressed as one of the leading therapeutic goals in T2D, with stringent targets must be achieved (71). This issue has also been recently reviewed by De Block et. that emphasized the importance of reaching adequate glycemic control and body weight loss as a common goal to improve the cardiovascular safety of these individuals. For this purpose, they reviewed the role of high-dose GLP-1RAs, such as Liraglutide 3 mg, Semaglutide 2 and 2.4 mg, Dulaglutide 3 and 4.5 mg, TZP, and the dual agonist glucagon/GLP-1 Cotadutide (72).

Interestingly, the results of the SURPASS-5 tell us information about the impressive weight loss observed with TZP in insulin users. A decline in insulin dose requirement could explain weight reduction after intensification with the dual agonist in this setting. However, as observed in the SURPASS-5, daily insulin dose remained similar in participants randomized to TZP 5 and 10 mg and was mildly reduced in those assuming TZP 15 mg. Therefore, the contribution of daily insulin dose reduction to weight loss appeared almost negligible, and the mechanisms underlying this effect are not completely understood. However, they could be attributable to the GIP component of TZP that exerts some extra glycemic synergistic effects with GLP-1RA and some others over GLP-1RA. As an example, in experimental conditions with animal models (GLP-1R-null), TZP reduced appetite and food intake, liver glycogenolysis and gluconeogenesis, improved insulin sensibility (assessed by euglycemic clamp) and glucose disposal in white and brown adipose tissue, skeletal muscle, with body weight-dependent and independent effects (73). These actions resulted in a significant reduction in body weight, corresponding to liver and adipose tissue weight restrictions (73). These findings suggest that GIP but not GLP-1RA may improve glucose disposal by ameliorating peripheral insulin sensibility and glucose utilization, while GIP added on to GLP-1RA may reduce food intake with a combined effect resulting in improvement of glucose control and weight loss.

## Conclusion

TZP appears a potent tool to improve glucose control without increasing hypoglycemic risk in poorly controlled T2D treated with basal insulin with or without other antihyperglycemic oral agents. Its impressive effect on body weight loss, despite background therapy, may be an important resource also for improving weight management in a usually difficult-to-treat cluster of patients.

## Data availability statement

The raw data supporting the conclusions of this article will be made available by the authors, on a reasonable request.

## Author contributions

GL conceived the systematic review and meta-analysis. GL and ADT elaborated the search strategy. GL and ADT developed the selection criteria and assessed the risk of bias in included studies. GL provided statistical expertise. GL and ADT drafted the manuscript. GL, ADT, OD, VDG, GP, GDP, VAG, EJ, EG, CS, and VT read the text, provided feedback, and approved the manuscript.

## Conflict of interest

The authors declare that the research was conducted in the absence of any commercial or financial relationships that could be construed as a potential conflict of interest.

## Publisher’s note

All claims expressed in this article are solely those of the authors and do not necessarily represent those of their affiliated organizations, or those of the publisher, the editors and the reviewers. Any product that may be evaluated in this article, or claim that may be made by its manufacturer, is not guaranteed or endorsed by the publisher.

## Abbreviations

DPP-IV, Dipeptidyl-Peptidase 4; FPG, Fasting Plasma Glucose; GIP, Gastric Inhibitory Polypeptide/Glucose-dependent Insulinotropic Polypeptide; GIPR, GIP Receptor; GLP-1R, GLP-1 Receptor; GLP-1Ras, GLP-1 receptor agonists; GLP-1, Glucagon-Like Peptide-1; RCTs, Randomized Clinical Trials; RR, Risk Ratio; TZP, Tirzepatide; T2D, Type 2 Diabetes.
